# Pulmonary Embolism in Patients Admitted With Peripartum Cardiomyopathy: Prevalence, Predictors, and Associated In-Hospital Adverse Events

**DOI:** 10.7759/cureus.60953

**Published:** 2024-05-23

**Authors:** Omar Elkattawy, Casey A Hamlet, Ryan Dikdan, Omar Mohamed, Thomas J Lee, Aysha Hussain, Sherif Elkattawy, Felix Afriyie, Afif Hossain, Julius M Gardin

**Affiliations:** 1 Internal Medicine, Rutgers University New Jersey Medical School, Newark, USA; 2 Medicine, Saint Barnabas Medical Center, Livingston, USA; 3 Cardiology, St. Joseph’s University Medical Center, Paterson, USA

**Keywords:** cardiology research, cardiology, cardio-obstetrics, peripartum cardiomyopathy, pulmonary embolism

## Abstract

Introduction

Peripartum cardiomyopathy (PPCM) is defined as an idiopathic left ventricular failure with reduced ejection fraction (EF <45%) that affects women in the last month of pregnancy or in the months after giving birth. The pathophysiology remains elusive, resulting in complications with varied severity; one of the most concerning complications is thromboembolism, specifically pulmonary embolism (PE). The purpose of this study was to characterize and evaluate the real-world prevalence, predictors, and outcomes of PE in PPCM.

Methods

The data were derived from the National Inpatient Sample (NIS) database from January 2016 to December 2019. The primary outcomes assessed were baseline and hospital admission characteristics and comorbidities for patients with PPCM with or without PE. Outcomes for PPCM patients with PE and predictors of mortality for PPCM were also analyzed.

Results

PE developed in 105 of 4,582 patients with PPCM (2.3%). Patients with PPCM and PE had longer hospital stays (10.86 days ± 1.4 vs. 5.73 ± 0.2 days, p = 0.001) and total charges ($169,487 ± $39,628 vs. $86,116 ± $3,700, p = 0.001). Patients with PE had a higher burden of coagulopathy (13.3% vs. 3.0%, p = 0.01), intracardiac thrombus (6.7% vs. 1.6%, p = 0.01), and iron deficiency anemia (21.0% vs. 12.6%, p = 0.01). Patients without PE were found to have a higher burden of preeclampsia (14.7% vs. 1.9%, p = 0.01) and obstructive sleep apnea (5.4% vs. 1.0%, p = 0.045). Predictors of mortality in patients with PPCM included cardiogenic shock (aOR 13.42, 95% CI 7.50-24.03, p = 0.05), PE (aOR 6.60, 95% CI 2.506-17.39, p = 0.05), non-ST-elevation myocardial infarction (NSTEMI; aOR 3.57, 95% CI 1.35-9.44, p = 0.05), chronic kidney disease (aOR 3.23, 95% CI 1.68-6.22, p = 0.05), and atrial fibrillation (aOR 2.57; 95% CI 1.25-5.30, p = 0.05).

Conclusion

Although an uncommon complication, PE in PPCM demonstrates an association with higher mortality and financial burden. Along with PE, we found predictors of mortality in PPCM to include atrial fibrillation, NSTEMI, chronic kidney disease, and cardiogenic shock.

## Introduction

Peripartum cardiomyopathy (PPCM) is defined as an idiopathic left ventricular (LV) failure with reduced ejection fraction (EF <45%) that affects women in the last month of pregnancy or the months after giving birth [1]. The pathophysiology remains elusive, but more recent studies have introduced the vasculo-hormonal hypothesis, which suggests PPCM can be attributed to elevated prolactin and sFlt1 [2]. Vasoinhibin is a metabolite of prolactin and damages endothelial cells, while sFlt1 functions as a VEGF inhibitor, resulting in further dysfunction and apoptosis of endothelial cells [3]. These simultaneous deleterious processes compromise vascular integrity and lead to cardiomyocyte damage. The diagnosis of PPCM may be difficult, mainly because the presenting symptoms, such as shortness of breath, orthopnea, and edema, are similar to those of a normal pregnancy [4]. A definitive diagnosis can be made with an echocardiogram showing LV systolic dysfunction in a patient with no other source of heart failure [4]. An echocardiogram is an obligatory part of the PPCM workup to rule out an LV thrombus as soon as possible [5]. High levels of brain natriuretic peptide, as well as electrocardiographic abnormalities identical to those in acute heart failure, can further support a diagnosis of PPCM [4]. The primary aim of the study was to assess whether or not there is a difference in outcomes (mortality, in-hospital complications, length of stay, and total charges) between the cohort of patients with PPCM and pulmonary embolism (PE) vs. patients with PPCM without PE. We also analyzed the independent predictors of mortality among patients admitted with PPCM.

## Materials and methods

Data acquisition

This is a retrospective database study of the National Inpatient Sample (NIS) database. The NIS is part of the Healthcare Cost and Utilization Project (HCUP) set forth by the Agency for Healthcare Research and Quality. It utilizes the International Classification of Diseases, Tenth Revision, Clinical Modification (ICD-10-CM) codes for diagnosis and procedures. The data set was utilized to examine the data of patients admitted between the years 2016 and 2019. Encounters with a primary diagnosis of PPCM were selected using ICD-10 code O90.3. This cohort of patients was further divided into patients who developed PE versus patients who did not develop this complication. Adult patients ≥18 years old were included. We abstracted data from 4,730 charts, excluded 148, and were left with 4,582 charts for analysis. IRB approval was not required as NIS provides de-identified information on patients.

Outcomes and variables

Patient baseline characteristics, such as age and race, were extracted. Comorbidities, hospital complications, mortality rates, disposition status, length of stay, and total charges were also analyzed. The primary aim of the study was to assess whether or not there is a difference in outcomes (mortality, in-hospital complications, length of stay, and total charges) between the cohort of patients with PPCM and PE vs. patients with PPCM without PE. We also analyzed the independent predictors of mortality among patients admitted with PPCM.

Statistical analysis

Categorical values were analyzed via Pearson Chi-square analysis, and continuous variables were analyzed via an independent Student’s t-test. Logistic regression was performed to generate odds ratios with 95% CIs to assess predictors of mortality in women with PPCM. We also used logistic regression to assess the independent association of PE with outcomes, taking into account confounders such as age, race, and comorbidities. A p-value of <0.05 was considered statistically significant. All analyses were completed using IBM SPSS Statistics for Windows, Version 29.0 (Released 2022; IBM Corp., Armonk, NY, USA).

## Results

Our cohort contained 4,582 patients with PPCM, 105 of whom had developed PE (2.3%). A statistical analysis of baseline characteristics is summarized in Table 1. On average, patients without PE were older in age than patients who developed PE (32 ± 7.2 vs. 30 ± 6.8, p = 0.003). There was no significant association between race and PE status. The disposition of patients after discharge differed significantly between those with and without PE, with more PE patients being transferred to skilled nursing facilities and other care facilities (7 (6.7%) vs. 85 (1.8%), p = 0.001).

**Table 1:** Baseline characteristics of the study population of PPCM patients with and without PE The data are presented as n and percentages. p-Values are considered significant at <0.05. PE, pulmonary embolism; PPCM, peripartum cardiomyopathy

Variable	No PE	PE	p-Value
Average age at admission	32 ± 7.2	30 ± 6.8	0.003
Post-hospital discharge	-	-	0.001
Routine	3,762 (81.4%)	71 (67.6%)	-
Transfer to a short-term hospital	214 (4.6%)	5 (4.8%)	-
Transfer other (includes skilled nursing facility, intermediate care facility, and other types of facility)	85 (1.8%)	7 (6.7%)	-
Home health care	404 (8.7%)	14 (13.3%)	-
Against medical advice	98 (2.1%)	2 (1.9%)	-
Died in hospital	61 (1.3%)	6 (5.7%)	-
Discharged/transferred to court/law enforcement	0 (0)	0 (0)	-
Discharged alive, destination unknown	0 (0)	0 (0)	-
Race	-	-	0.896
White	1,684 (37.6%)	39 (37.9%)	-
Black	1,986 (44.3%)	48 (46.6%)	-
Hispanic	488 (10.9%)	11 (10.7%)	-
Asian or Pacific Islander	124 (2.8%)	2 (1.9%)	-
Native American	54 (1.2%)	0 (0)	-
Other	144 (3.2%)	3 (2.9%)	-

The results of the univariate analysis showing the associations between several comorbidities and PE status in PPCM are depicted in Table 2. Patients with PE had a higher burden of coagulopathy (14 (13.3%) vs. 137 (3.0%), p = 0.01), intracardiac thrombus (7 (6.7%) vs. 73 (1.6%), p = 0.01), and iron deficiency anemia (22 (21.0%) vs. 581 (12.6%), p = 0.01). Interestingly, preeclampsia (678 (14.7%) vs. 2 (1.9%), p = 0.01) and obstructive sleep apnea (248 (5.4%) vs. 1 (1.0%), p = 0.045) were found to be higher in patients without PE than in their counterparts with PE.

**Table 2:** Analysis of comorbidities of PPCM patients stratified by PE status The data are presented as n and percentages. p-Values are considered significant at <0.05. COPD, chronic obstructive sleep apnea; PE, pulmonary embolism; PPCM, peripartum cardiomyopathy

Variable	No PE	PE	p-Value
COPD	786 (17%)	13 (12.4%)	0.212
Coagulopathy	137 (3%)	14 (13.3%)	0.001
Cerebrovascular disease	26 (0.6%)	0 (0)	0.441
Type 2 diabetes mellitus	457 (9.9%)	5 (4.8%)	0.081
Hypertension	333 (7.2%)	12 (11.4%)	0.099
Alcohol use disorder	41 (0.9%)	2 (1.9%)	0.277
Liver disease	174 (3.8%)	5 (4.8%)	0.596
Peripheral vascular disease	4 (0.1%)	0 (0)	0.763
Atrial fibrillation	256 (5.5%)	2 (1.9%)	0.105
Hypothyroidism	251 (5.4%)	2 (1.9%)	0.113
HIV	3 (0.1%)	0 (0)	0.794
Coronary artery disease	142 (3.1%)	4 (3.8%)	0.665
Intracardiac thrombus	73 (1.6%)	7 (6.7%)	0.001
Pulmonary hypertension	530 (11.5%)	12 (11.4%)	0.992
Tobacco use disorder	32 (0.7%)	1 (1%)	0.751
Obstructive sleep apnea	248 (5.4%)	1 (1%)	0.045
Iron deficiency anemia	581 (12.6%)	22 (21%)	0.011
Chronic kidney disease	373 (8.1%)	6 (5.7%)	0.38
Gestational diabetes	137 (3%)	1 (1%)	0.226
Gestational hypertension	197 (4.3%)	5 (4.8%)	0.801
Preeclampsia	678 (14.7%)	2 (1.9%)	0.001
Cocaine use disorder	57 (1.2%)	2 (1.9%)	0.539
Opioid use disorder	100 (2.2%)	4 (3.8%)	0.255
Obesity	507 (11%)	13 (12.4%)	0.646

A summary of unadjusted analyses of outcomes of PPCM patients with and without PE is included in Table 3. Univariate analyses show that PE was associated with higher rates of mortality (6 (5.7%) vs. 61 (1.3%), p = 0.001), vasopressor use (4 (3.8%) vs. 48 (1.0%), p = 0.007), cardiac arrest (6 (5.7%) vs. 82 (1.8%), p = 0.003), and ventricular tachycardia (13 (12.4%) vs. 296 (6.4%), p = 0.014). Independent sample t-test analysis showed that the length of hospital stay was significantly longer in those with PE compared to those without PE (10.86 days vs. 5.73 days, p = 0.001). Overall hospitalization costs were also significantly higher in the PE group ($169,487 vs. $86,116, p = 0.001).

**Table 3:** Analysis of outcomes for PPCM patients stratified by PE status The data are presented as n and percentages. p-Values are considered significant at <0.05. HELLP, hemolysis, elevated liver enzymes, and low platelets syndrome; NSTEMI, non-ST-elevation myocardial infarction; PE, pulmonary embolism; PPCM, peripartum cardiomyopathy; STEM, ST-elevation myocardial infarction

Variable	No PE	PE	p-Value
Died at discharge	61 (1.3%)	6 (5.7%)	0.001
Length of stay (days)	5.73	10.86	0.001
Total charges ($)	86,116	169,487	0.001
Permanent pacemaker implantation	5 (0.1%)	0 (0)	0.736
Cardiogenic shock	267 (5.8%)	10 (9.5%)	0.106
Vasopressor use	48 (1%)	4 (3.8%)	0.007
Intra-aortic balloon pump	83 (1.8%)	4 (3.8%)	0.129
Mechanical ventilation	153 (3.3%)	0 (0)	0.058
Ventricular fibrillation	92 (2%)	3 (2.9%)	0.531
Ventricular tachycardia	296 (6.4%)	13 (12.4%)	0.014
Atrioventricular block	91 (2%)	2 (1.9%)	0.963
Tricuspid regurgitation	67 (1.4%)	2 (1.9%)	0.7
Left heart catheterization	139 (3%)	2 (1.9%)	0.512
Right heart catheterization	229 (5%)	9 (8.6%)	0.093
Cardiac arrest	82 (1.8%)	6 (5.7%)	0.003
Eclampsia	71 (1.5%)	1 (1%)	0.63
HELLP syndrome	41 (0.9%)	0 (0)	0.333
Shock after delivery	70 (1.5%)	0 (0)	0.204
Postpartum hemorrhage	159 (3.4%)	5 (4.8%)	0.463
STEMI	9 (0.2%)	1 (1%)	0.095
NSTEMI	162 (3.5%)	0 (0)	0.051

**Figure 1:**
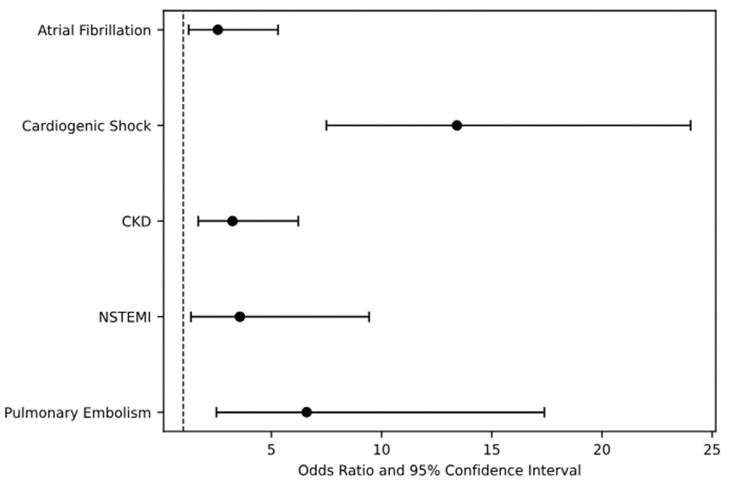
Multivariate logistic regression of predictors of mortality in PPCM patients CKD, chronic kidney disease; NSTEMI, non-ST-elevation myocardial infarction; PPCM, peripartum cardiomyopathy

Multivariate logistic regression was used to assess the predictors of mortality in patients with PPCM, as shown in Figure 1. Predictors of mortality included cardiogenic shock (aOR 13.42, 95% CI 7.50-24.03, p < 0.05), PE (aOR 6.60, 95% CI 2.506-17.39, p < 0.05), non-ST-elevation myocardial infarction (NSTEMI; aOR 3.57, 95% CI 1.35-9.44, p < 0.05), CKD (aOR 3.23, 95% CI 1.68-6.22, p < 0.05), and atrial fibrillation (aOR 2.57; 95% CI 1.246-5.30, p < 0.05). We also used multivariate logistic regression factoring in age, race, and comorbidities to evaluate the independent association of PE with outcomes. PE was independently associated with cardiac arrest (aOR 3.39, 95% CI 1.18-9.81, p < 0.05).

## Discussion

Our analysis of 4,582 patients with PPCM showed the prevalence of PE to be 2.3%. Although this incidence is relatively low, the adverse effects of PE in PPCM are severe and can be fatal, necessitating close monitoring of these patients. The vast majority of previous publications about PE in PPCM are comprised of case studies; to our knowledge, there are no prevalence or incidence data with which to compare our findings. In our study, PPCM patients with PE had a longer length of hospital stay and increased hospitalization costs compared to PPCM patients without PE. PPCM and concurrent PE were associated with higher rates of inpatient mortality, ventricular tachycardia, and cardiac arrest, as well as the use of vasopressors. Factors that affected mortality for all patients with PPCM included atrial fibrillation, CKD, NSTEMI, PE, and cardiogenic shock.

The pathophysiology of PPCM is not well understood, but it is thought to be mediated by the hormonal changes of pregnancy, with genetic predisposition postulated to play a role [5]. The antiangiogenic and cardiotoxic effects of the N-terminal 16 kDa fragment of prolactin, later named vasoinhibin, have been well documented, and the elevated prolactin in the peripartum period makes these patients susceptible to endothelial cell damage and ventricular wall angiogenesis [2,6,7]. Newer studies focusing on the role of genetics in PPCM suggest that patients who are carriers of genetic mutations that cause cardiomyopathies may be more prone to developing PPCM when they become pregnant [8].

PPCM is associated with similar complications to those of other cardiomyopathies; these patients are at risk for progression to severe heart failure, cardiogenic shock, cardiac arrhythmias, and thromboembolism [2]. Hypokinesis of the left ventricle and subsequent blood stasis within the heart predispose these patients to thrombosis. This condition, in addition to the baseline hypercoagulability of pregnancy and the early postpartum period, is consistent with our findings that patients with PE had higher burdens of both coagulopathy and intracardiac thrombosis.

Management of PPCM is similar to that of other causes of heart failure with reduced EF and includes loop diuretics, beta-blockers, and sometimes, inotropes [4]. Unfortunately, the similarities between the presentation of PPCM and normal pregnancy can lead to a delay in diagnosis and an increased risk of adverse events associated with PPCM. One such adverse event is the development of PE. The pathophysiology behind this association is likely due to a combination of factors such as hemostasis, resulting from ventricular dysfunction; postpartum immobility, especially during cesarean section recovery; and the hypercoagulability of pregnancy [8].

For both prophylactic and therapeutic purposes, anticoagulation with low-molecular-weight heparin and unfractionated heparin is encouraged, but recommendations about the dosage and duration are not well defined [2,9]. The American Heart Association suggests temporary anticoagulation in patients with LVEF ≤30%, while the European Society of Cardiology is more conservative, recommending anticoagulation when LVEF is ≤35% [2]. Postnatally, warfarin or heparin can be used for anticoagulation because neither is excreted in breast milk [4]. There is a lack of a more specific protocol pertaining to the initiation and cessation of treatment; however, most patients will not have to be anticoagulated indefinitely as PPCM has a relatively high rate of recovery compared to other dilated cardiomyopathies [4].

Our findings also showed a higher incidence (12.4% vs. 6.4%) of ventricular tachycardia in PPCM patients with PE, which is consistent with previous studies [2,4]. PPCM patients are at a significantly higher risk for sudden cardiac death due to arrhythmia, with ventricular tachycardia being the most common arrhythmogenic cause. Newer research focuses on optimal management for patients with impaired LV systolic function [10]. Currently, a wearable cardioverter/defibrillator is first-line for the detection and treatment of life-threatening arrhythmias in PPCM, and intracardiac device or cardiac resynchronization therapy implantation is indicated when optimal medical management and less invasive methods are insufficient.

The present study also demonstrated a significantly higher incidence (5.7% vs. 1.8%) of cardiac arrest in PPCM patients with PE. Cardiac arrest as a major adverse event of PPCM has been well defined in prior studies [2,11,12]. Management of cardiac arrest in PPCM is essentially unchanged from standard resuscitation protocol except if the patient is antepartum and the uterus is palpated at or above the umbilicus, in which case supine positioning will compromise resuscitative efforts by compressing the aorta and inferior vena cava. In this case, manual left uterine displacement is accomplished by cupping the uterus and pushing it upward and leftward to relieve aortocaval compression [2]. Left uterine displacement should be performed for the entirety of resuscitation.

Our study also found that PPCM patients with PE had a lower incidence of preeclampsia (1.9% vs. 14.7%). A retrospective nationwide survey conducted by Kamiya et al. found that hypertensive disorders of pregnancy in PPCM were associated with shorter hospital stays and a higher LVEF [13]. Another study by Haghikia et al. found that pregnancy-associated hypertension was associated with LVEF improvement at six-month follow-up [14]. Further research is needed to explore this association. Our analysis also showed that cardiogenic shock was a significant predictor of mortality in PPCM patients. It is recommended that PPCM patients in cardiogenic shock be transferred to a facility that has mechanical circulatory support and ventricular assist devices [8]. Generally, norepinephrine is the vasopressor of choice, while the use of beta-adrenergic agonists in PPCM is generally avoided, as experimental studies have demonstrated irreversible cardiotoxicity and heart failure exacerbations [8,15,16].

Strengths of our studies include the fact that our sample size remains the highest ever reported using data drawn from a database registry. To our knowledge, the only studies addressing PE in PPCM have been case reports. Our study is the first to provide a comprehensive assessment of the risk of PE in PPCM and its incidence and prevalence, thus addressing an unmet need for further research.

Our study is limited by the fact that it is an observational study; thus, we can only infer association and not causation. Furthermore, the NIS is an ICD-10 administrative database, thus prone to human coding errors. In our study, we did not sub-stratify PE into low-risk, sub-massive, and massive PE. Future studies should utilize ICD-10 codes to further sub-stratify PE into PE with vs. without acute cor pulmonale. In addition, our study did not examine which treatments were given for the PE (anticoagulation vs. lysis vs. thrombectomy).

## Conclusions

Physicians should maintain a high index of suspicion for PE in patients with PPCM, especially in patients with risk factors and associated comorbidities. Heightened awareness of this cardiovascular emergency in peripartum women will not only reduce costs and length of stay in the hospital but also reduce diagnostic delay, adverse events, and death. Future studies should investigate the association between diagnostic delay and morbidity of PE in PPCM patients, further demonstrating the importance of early recognition and management of this condition.
